# Amoxicillin effect on bacterial load in group A streptococcal pharyngitis: comparison of single and multiple daily dosage regimens

**DOI:** 10.1186/s12887-019-1582-8

**Published:** 2019-06-21

**Authors:** Akihiro Nakao, Ken Hisata, Makoto Fujimori, Nobuaki Matsunaga, Mitsutaka Komatsu, Toshiaki Shimizu

**Affiliations:** 1 Department of Pediatrics, Faculty of Medicine, Juntendo University, 2-1-1 Hongo, Bunkyo-ku, Tokyo, 113-8421 Japan; 2 Fujimori Children’s Clinic, 1499-1 Amadocho, Hanamigawa-ku, Chiba-shi, Chiba 262-0043 Japan

**Keywords:** Group a streptococcus, *Streptococcus pyogenes*, Amoxicillin, Pharyngitis, Bacterial load, Quantification

## Abstract

**Background:**

Culture tests have demonstrated that once-daily administration of amoxicillin may be effective in the treatment of group A streptococcal (GAS) pharyngitis. However, culture methods do not allow accurate assessments of bacterial load changes because of the suppressive effect of the antibiotic on bacterial growth. In this study, we used real-time PCR to compare the effectiveness of once-daily and multiple-daily amoxicillin treatment for pediatric patients with GAS pharyngitis.

**Methods:**

The subjects were children (≧3 years of age) diagnosed with GAS pharyngitis. Amoxicillin was administered at a dose of 40–50 mg/kg/day, divided into one (QD), two (BID), or three (TID) daily doses, for 10 days. Throat swabs were collected before treatment (visit 1), 1 to 3 days after treatment (visit 2), and 9 to 11 days after treatment (visit 3), and GAS copies were quantified by real-time PCR. The main compared parameters were the rate of negative PCR results and the number of GAS determined by PCR in throat swabs between each regimen.

**Results:**

Samples were collected from 34 patients (QD, 12; BID, 15; TID, 7) at visit 1, 32 patients (QD, 11; BID, 14; TID, 7) at visit 2, and 25 patients (QD, 7; BID, 11; TID, 7) at visit 3. The rates of negative PCR result for QD, BID, and TID regimens were 18.2, 0, and 14.3% at visit 2, and 85.7, 72.7, and 85.7% at visit 3, respectively. The median values of bacterial load for QD, BID, and TID groups at visit 1 were 1.4 × 10^6^, 8.2 × 10^5^, and 5.4 × 10^5^ copies/μL. At visit 2, they comprised 3.8 × 10^3^, 1.1 × 10^3^, and 2.8 × 10^3^ copies/μL, respectively, whereas at visit 3, GAS copies were mostly undetectable. There was no statistical difference in the negative results and median value of GAS copies between regimens at any stage.

**Conclusions:**

Our results obtained by a molecular biology approach indicated that the QD regimen was as effective in eradicating GAS infection as BID or TID.

**Trial registration:**

UMIN000036083 / March 12, 2019.

## Background

Group A beta-hemolytic Streptococcus (GAS) causes a wide variety of clinical conditions: upper respiratory tract infections, skin and soft tissue infections, and toxic-shock syndrome, as well as non-pyogenic secondary diseases, such as acute glomerulonephritis and rheumatic fever [1]. The purpose of antibiotic therapy of GAS infection is to reduce acute phase symptoms and prevent pyogenic complications and rheumatic fever [2]. For this reason, rapid antigen and culture tests are recommended for children over the age of three, who are suspected to have a GAS infection [3]. If the pathogen is detected by these tests, then an appropriate antibiotic treatment is necessary: the administration of penicillin class drugs is recommended according to the guidelines [3]. Since this treatment was first introduced in 1950s, it has been used by many clinicians, and its widespread application has contributed greatly to the prevention of rheumatic fever [4].

Penicillin class drugs are commonly administered several times a day. However, the regimen of once-daily amoxicillin in GAS pharyngitis has been studied for over 20 years as the efficacy of such treatment has been shown to be equivalent to that of the multiple-daily regimen [5–9]. In these studies, the use of the single dose regimen has been advocated on the basis of the clinical course after the treatment, culture test results, and adverse events. It is certainly very important to evaluate these parameters to assess the therapeutic effects. However, culture after treatment does not accurately report the presence of pathogens because the suppressive effect of antibiotics influences its robustness. In this situation, false-negative results may be obtained. Homme et al. reported that PCR is more sensitive than culture methods in comparing positive rates after antibiotic treatment for GAS pharyngitis [10]. Thus, we analyzed the presence of pathogens and quantitative changes in bacterial load using a molecular method to compare the effectiveness of the antibiotic regimens. The evaluation of antibiotic therapy needs a more microbiologically precise method, although it is possible that GAS copies detected after the treatment are from bacteria already damaged by antibiotics.

In this study, we compared the effects of once-daily and multiple-daily administrations of oral amoxicillin on bacterial load in throat swabs collected in GAS pharyngitis cases by using real-time PCR at three different points. This is the first report evaluating bacterial loads from GAS lesion areas by genetic methods.

## Methods

### Study design and patients

The study subjects were children aged older than 3 years, who visited pediatric department at two medical institutions in Japan between October 2015 and September 2016 and were suspected of having acute pharyngeal tonsillitis due to a GAS infection. Clinical diagnoses were made on the basis of reference symptoms, such as fever, sore throat, malaise, and headache with acute onset, whereas the physical findings that suggested the infection included prominent pharyngeal redness, tonsil swelling with exudate, and cervical lymphadenopathy [11]. The study subjects underwent rapid antigen testing with ImunoAce StrepA (Tauns, Shizuoka, Japan), using a throat swab and isolation culture tests, in which GAS was detected. Patients with a history of penicillin allergies or those who received antibacterial drugs within the previous 4 weeks were excluded from this study. For all subjects, amoxicillin was administered at a dose of 40 to 50 mg/kg/day with an upper limit of 1000 mg/day, divided into one (QD), two (BID), or three (TID) daily doses, for 10 days. Patient families determined daily times of antibiotic dose administration according to their lifestyle. No antimicrobial agent other than amoxicillin was given to patients enrolled in this study. The main outcomes were the negative rate and a number of GAS copies determined by PCR in throat swabs collected after the start of treatment. Differences in these parameters were compared between QD, BID, and TID regimens. The clinical course after the treatment was examined during outpatient visits at later dates. All subjects were educated as to the symptoms of relapse and complications. They were also instructed to come for re-examination if any of these symptoms were suspected.

### Sampling and detection of GAS

Throat swab samples were collected using FLOQ double swabs (Copan, Brescia, Italy) at three time points: during visit 1 (before the start of the treatment), visit 2 (1 to 3 days after the treatment), and visit 3 (9 to 11 days after the treatment). One swab sample was seeded on blood agar medium and cultured at 36 °C for 24–48 h to check for the development of colonies exhibiting β hemolysis. Then, the latex agglutination test (Strept LA, DENKA SEIKEN Co., Ltd., Tokyo, Japan) was used to identify GAS. The other swab was cryopreserved at − 80 °C until DNA extraction.

### DNA extraction and quantitative PCR

Bacterial load was calculated from the solution of throat swab samples. After stored throat swab samples were treated with achromopeptidase, DNA was extracted using a QIAamp DNA Mini Kit (QIAGEN, Hilden Germany). Real-time PCR was performed using primers, a probe targeting s*pe* B, as described in a previous report [12], and a TaqMan Fast Advanced Master Mix (Thermo Fisher Scientific, MA, USA). As positive control, DNA of *Streptococcus pyogenes* ATCC BAA-572 was used. Plasmid DNA, in which a detection region was introduced by the TA cloning method, was used as standard DNA. Correct targeting of the detection region to plasmid DNA was confirmed by sequencing using an Applied Biosystems 3130 Genetic Analyzer (Thermo Fisher Scientific, MA, USA). In order to prepare a calibration curve, the number of DNA copies per μL of standard DNA solution was calculated in advance. The detection limit of quantitative real-time PCR was defined as 10^2^ copies per μL, corresponding to a threshold cycle value of 35 on the calibration curve.

### Data analysis

Analyses were conducted using Prism 8.1.1 (GraphPad Software, Inc., San Diego, CA, USA). Patient population demographics, clinical symptoms and signs, and the rate of negative PCR results between treatment groups were compared by the Student’s unpaired *t*-test and Fisher’s exact test. The Mann-Whitney U-test was used to compare the median of bacterial load between QD and BID or TID, respectively. All statistical analyses were conducted with a significance level of α = 0.05 (*P* < 0.05).

### Ethical approval

This study was approved by the Ethics Committee of the Tokyo Metropolitan Health Public Corporation Toshima Hospital. For all patients, written informed consent was provided by a parent and/or legal proxy.

## Results

Fifty-one patients were suspected to have GAS pharyngitis clinically and were positive in the rapid antigen test. In 12 of these patients, GAS was not detected using the culture test, whereas 5 other patients did not agree to participate in this study. Of the remaining 34 subjects in this study, 12, 15, and 7 took amoxicillin using QD, BID, and TID regimens. All patients confirmed that they had taken antibiotics according to the respective regimens at visit 3. The QD group was older and comprised significantly more boys than the BID group (Table 1).

**Table 1:** Characteristics of patients included in the study

	QD *n* = 12 (%)	BID *n* = 15 (%)	*P*-value	TID *n* = 7 (%)	*P*-value
Age, years^a^	7.3 ± 3.1	5.0 ± 1.4	**0.02**	7.7 ± 1.3	0.71
Male sex	12 (100)	10 (66.7)	**0.047**	6 (85.7)	0.37
Symptoms					
Fever^b^	12 (100)	14 (93.3)	1.00	7 (100)	1.00
Absence of cough	9 (75.0)	13 (86.6)	0.63	4 (57.1)	0.62
Sore throat	7 (58.3)	9 (60.0)	1.00	6 (85.7)	0.33
Headache	6 (50.0)	5 (33.3)	0.45	3 (42.9)	1.00
Abdominal pain	0 (0)	4 (26.7)	0.11	2 (28.6)	0.12
Nausea	1 (8.3)	3 (20.0)	0.61	1 (14.3)	1.00
Signs					
Lymphadenopathy	9 (75.0)	12 (80.0)	1.00	6 (85.7)	1.00
Tonsillar swelling	8 (66.7)	13 (86.7)	0.36	4 (57.1)	1.00
Skin rash	2 (16.7)	3 (20.0)	1.00	0 (0)	0.51

*Abbreviations*: *QD* quaque die, *BID* bis in die, *TID* ter in die

The unpaired Student’s *t*-test was used to compare age, and the Fisher’s exact test was used to evaluate possible differences in the distribution of sex, symptoms, and clinical signs between QD and BID or TID, respectively

^a^Age is presented as the mean ± standard deviation

^b^Fever was defined as a temperature above 38 °C by axillary measurement

Specimens from all 34 patients were collected during visit 1. During visit 2 and 3, additional samples were collected from 32 and 25 patients, respectively. The results of PCR and culture test are shown in Table 2. At visit 2, 95% confidence intervals (CIs) of the negative PCR result rate using PCR were 3.2 to 47.7, 0 to 21.5, and 0.7 to 51.3 for QD, BID, and TID, respectively. At visit 3, the corresponding CIs were 48.7 to 99.3, 43.4 to 90.3, and 48.7 to 99.3. When the culture test was used, only one patient was positive at visit 2 and visit 3. All patients exhibited symptomatic improvement after antimicrobial treatment, with no recurrence or complications such as rheumatic fever, including patients positive for GAS at visit 3.

**Table 2:** PCR and culture tests of pharyngeal swabs

			Visit 1 *n* = 34	Visit 2 *n* = 32	Visit 3 *n* = 25
qPCR	Negative (%)	QD	0 / 12 (0)	2 / 11 (18.2)	6 / 7 (85.7)
		BID	0 / 15 (0)	0 / 14 (0)	8 / 11 (72.7)
		TID	0 / 7 (0)	1 / 7 (14.3)	6 / 7 (85.7)
Culture	Negative (%)	QD	0 / 12 (0)	11 / 11 (100)	7 / 7 (100)
		BID	0 / 15 (0)	14 / 14 (100)	10 / 11 (90.9)
		TID	0 / 7 (0)	6 / 7 (85.7)	7 / 7 (100)

*Abbreviations*: *QD* quaque die, *BID* bis in die, *TID* ter in die, *qPCR* quantitative polymerase chain reaction

The Fisher’s exact test was used to compare the negative rate between QD and BID or TID, respectively. *P* values were 0.18 and 1.00 at visit 2 between QD and BID and QD and TID, whereas at visit 3, *P* value was both of these comparisons was 1.00.

The median values of bacterial load for QD, BID, and TID groups at visit 1 were 1.4 × 10^6^, 8.2 × 10^5^, and 5.4 × 10^5^ copies/μL and at visit 2 were 3.8 × 10^3^, 1.1 × 10^3^, and 2.8 × 10^3^ copies/μL, respectively, whereas at visit 3, GAS copies were mostly undetectable **(**Table 3**).** The differences in the median values between QD and BID regimens, and between QD and TID were not statistically significant at any stage of the experiment. The 95% CI values for the median of GAS copies at visit 1 were within a narrow range for all regimens. At visit 2, the interquartile range values were also relatively close for all groups, but 95% CI values had a very wide range, and the same tendency was observed at visit 3. GAS copies in the two patients that were culture positive after treatment were 6.9 × 10^4^ (TID group, visit 2) and 7.1 × 10^4^ copies/μL (BID group, visit 3), respectively.

**Table 3:** Quantitative analysis of GAS strains

	QD	BID	TID
Visit 1			
*n*	12	15	7
median, copies /μL	1.4 × 10^6^	8.2 × 10^5^	5.4 × 10^5^
[interquartile range]	[2.2 × 10^5^–4.3 × 10^6^]	[2.4 × 10^5^–1.6 × 10^6^]	[1.4 × 10^5^–2.2 × 10^6^]
95% confidence interval	2.2 × 10^5^, 4.3 × 10^6^	2.4 × 10^5^, 1.6 × 10^6^	1.0 × 10^5^, 6.1 × 10^6^
Visit 2			
*n*	11	14	7
median, copies /μL	3.8 × 10^3^	1.1 × 10^3^	2.8 × 10^3^
[interquartile range]	[2.7 × 10^2^–2.8 × 10^4^]	[5.2 × 10^2^–4.8 × 10^3^]	[1.8 × 10^2^–6.9 × 10^4^]
95% confidence interval	0.0, 1.2 × 10^5^	4.9 × 10^2^, 8.0 × 10^3^	0.0, 1.3 × 10^5^
Visit 3			
*n*	7	11	7
median copies /μL	0	0	0
[interquartile range]	[0.0–0.0]	[0.0–1.2 × 10^2^]	[0.0–0.0]
95% confidence interval	0.0, 9.0 × 10^3^	0.0, 1.7 × 10^4^	0.0, 2.8 × 10^3^

*Abbreviations*: *QD* quaque die, *BID* bis in die, *TID* ter in die

The Mann-Whitney U-test was used to compare the median of bacterial load between QD and BID or TID, respectively.

## Discussion

In order to assess the effectiveness of the antimicrobial therapy, it is very important to confirm the result of the culture test after the treatment. However, it is difficult to capture subtle changes in bacterial load by using the culture method, so it is necessary to evaluate it by a more accurate approach. This study is the first report in which changes in bacterial load before and after treatment were assessed using quantitative PCR.

Quantitative analysis of the samples collected before the antibiotic therapy revealed slightly higher initial bacterial load in the QD group compared to the values in BID and TID groups. We set the maximum dose for all subjects at 1000 mg per day, but there are no data to support the TID regimen with an upper limit of 1000 mg per day, therefore the blood levels might end up lower than the minimum inhibitory concentration (MIC) of amoxicillin for GAS. Nevertheless, there were no significant differences in the rate of negative PCR results and quantitative parameters between QD, BID, and TID at repeated visits after the antibiotic treatment. If we define a non-inferiority margin as 10%, following previous reports [7, 8], the rate of negative PCR results in QD group at visits 2 and 3 was within the upper 95% CI for BID and TID. The outcome of eradication following QD regimen was not inferior to that achieved after BID and TID regimens, however it should be noted that the 95% CI was very wide because of the small number of samples. For quantitative analysis, the bacterial load at visit 1 was similar among the groups, although significant differences were observed in the age and sex of the subjects. At visit 2, each regimen resulted in reduction to approximately 1/100 or more compared to the median values at visit 1. At visit 3, eradication of GAS was genetically confirmed in many cases. There were no significant differences between the results obtained after QD and after the other two regimens, but the number of samples was insufficient to indicate non-inferiority of the QD regimen.

Because the antibacterial effect of amoxicillin predominantly depends on the duration of its binding to the penicillin-binding proteins and the resulting inhibition of bacterial wall synthesis, it could be expected that administration of multiple doses would be more effective. We believe there are two reasons why there was no significant difference in the PCR results between QD and other regimens. One is the pharmacokinetics of amoxicillin: its absorption into the bloodstream and distribution to the respiratory tract after oral administration are very high [13, 14]. This is why amoxicillin is recommended as one of the most useful antimicrobial agents for respiratory infections, including GAS pharyngitis [15, 16]. The second reason is high susceptibility of GAS strains to amoxicillin. The MIC of penicillins for GAS is very low, and there have been no reports of antimicrobial resistance of GAS strains to the drugs of this class [17, 18]. Because of these two factors, amoxicillin achieves a high concentration in the pharyngeal lesion site, which remains above the MIC for a sufficient period of time even after QD regimen.

The guideline of the Infectious Diseases Society of America recommends a 10-days duration of treatment with amoxicillin for GAS pharyngitis [3]. However, the efficacy of shorter therapy has been also reported [19, 20]. In our study, 30 patients out of 32 tested at visit 2 and 5 out of 25 at visit 3 were PCR positive for GAS, respectively, so there is a concern about how efficiently GAS could be eradicated by a short treatment. It should be noted that positive PCR result does not necessarily mean treatment failure. More data should be accumulated before short regimen therapy may be recommended.

Although reducing the number of doses contributes to improved patient adherence [21], we do not recommend only QD regimen as the preferred and sufficient treatment for GAS pharyngitis. Designing dose regimens, including multiple administrations per day, which fit the patient’s wishes and lifestyle is very important to improve adherence [22]. In addition, the burden associated with an increase in the dosage per time should be observed carefully in pediatric patients. Clinicians should decide on the preferred number of dosages per day taking these factors into consideration.

## Conclusion

By using real-time PCR, we compared bacterial loads in samples collected from colonized lesions in patients with GAS pharyngitis following QD, BID, and TID amoxicillin treatment regimens. Our results obtained by the molecular biology approach indicated that the QD regimen was as effective in eradicating the infection as BID or TID regimens, although this may appear counterintuitive given the dynamics of the antibacterial effect of amoxicillin.

## Data Availability

The datasets obtained during the current study are available from the corresponding author on a reasonable request.

## Abbreviations

BID: Bis in die
CI: Confidence interval
GAS: Group A streptococcus
MIC: Minimum inhibitory concentration
QD: Quaque die
TID: Ter in die

## References

[1] Bryant AE, Stevens DL, Bennett JE, Dolin R, Blaser MJ (2015). Streptococcus pyogenes. Mandell, Douglas, and Bennett's principles and practice of infectious diseases.

[2] Gerber MA, Baltimore RS, Eaton CB, Gewitz M, Rowley AH, Shulman ST (2009). Prevention of rheumatic fever and diagnosis and treatment of acute streptococcal pharyngitis: a scientific statement from the American Heart Association rheumatic fever, endocarditis, and Kawasaki disease Committee of the Council on cardiovascular disease in the young, the interdisciplinary council on functional genomics and translational biology, and the interdisciplinary council on quality of care and outcomes research: endorsed by the American Academy of Pediatrics. Circulation.

[3] Shulman ST, Bisno AL, Clegg HW, Gerber MA, Kaplan EL, Lee G (2012). Clinical practice guideline for the diagnosis and management of group a streptococcal pharyngitis: 2012 update by the Infectious Diseases Society of America. Clin Infect Dis.

[4] Lee GM, Wessels MR (2006). Changing epidemiology of acute rheumatic fever in the United States. Clin Infect Dis.

[5] Shvartzman P, Tabenkin H, Rosentzwaig A, Dolginov F (1993). Treatment of streptococcal pharyngitis with amoxycillin once a day. BMJ.

[6] Feder HM, Gerber MA, Randolph MF, Stelmach PS, Kaplan EL (1999). Once-daily therapy for streptococcal pharyngitis with amoxicillin. Pediatrics.

[7] Clegg HW, Ryan AG, Dallas SD, Kaplan EL, Johnson DR, Norton HJ (2006). Treatment of streptococcal pharyngitis with once-daily compared with twice-daily amoxicillin: a noninferiority trial. Pediatr Infect Dis J.

[8] Lennon DR, Farrell E, Martin DR, Stewart JM (2008). Once-daily amoxicillin versus twice-daily penicillin V in group a beta-haemolytic streptococcal pharyngitis. Arch Dis Child.

[9] Schwartz RH, Kim D, Martin M, Pichichero ME (2015). A reappraisal of the minimum duration of antibiotic treatment before approval of return to school for children with streptococcal pharyngitis. Pediatr Infect Dis J.

[10] Homme JH, Greenwood CS, Cronk LB, Nyre LM, Uhl JR, Weaver AL (2018). Duration of group a Streptococcus PCR positivity following antibiotic treatment of pharyngitis. Diagn Microbiol Infect Dis.

[11] Sande L, Flores AR, Cherry J, Demmler-Harrison GJ, Kaplan SL, Steinbach WJ, Hotez PJ (2013). Group a, group C, group G Beta hemolytic streptococcal infections. Feigin and Cherry's textbook of pediatric infectious diseases.

[12] Dunne EM, Marshall JL, Baker CA, Manning J, Gonis G, Danchin MH (2013). Detection of group a streptococcal pharyngitis by quantitative PCR. BMC Infect Dis.

[13] Neely MN, Reed MD, Long SS, Pickering LK, Prober CG (2012). Pharmacokinetic–pharmacodynamic basis of optimal antibiotic therapy. Principles and practice of pediatric infectious diseases.

[14] Bradley JS, Sauberan JB, Long SS, Pickering LK, Prober CG (2012). Antimicrobial agents. Principles and practice of pediatric infectious diseases.

[15] Bradley JS, Byington CL, Shah SS, Alverson B, Carter ER, Harrison C (2011). The management of community-acquired pneumonia in infants and children older than 3 months of age: clinical practice guidelines by the Pediatric Infectious Diseases Society and the Infectious Diseases Society of America. Clin Infect Dis.

[16] Harris AM, Hicks LA (2016). Qaseem a, high value care task force of the American College of Physicians and for the Centers for Disease Control and Prevention. Appropriate antibiotic use for acute respiratory tract infection in adults: advice for high-value care from the American College of Physicians and the Centers for Disease Control and Prevention. Ann Intern Med.

[17] Brook I (2002). Antibacterial therapy for acute group a streptococcal pharyngotonsillitis: short-course versus traditional 10-day oral regimens. Paediatr Drugs.

[18] Casey JR, Pichichero ME (2005). Meta-analysis of short course antibiotic treatment for group a streptococcal tonsillopharyngitis. Pediatr Infect Dis J.

[19] Suzuki T, Kimura K, Suzuki H, Banno H, Jin W, Wachino J (2015). Have group a streptococci with reduced penicillin susceptibility emerged?. J Antimicrob Chemother.

[20] Cattoir V, Ferretti JJ, Stevens DL, Fischetti VA (2016). Mechanisms of antibiotic resistance. *Streptococcus pyogenes* basic biology to clinical manifestations.

[21] Claxton AJ, Cramer J, Pierce C (2001). A systematic review of the associations between dose regimens and medication compliance. Clin Ther.

[22] Osterberg L, Blaschke T (2005). Adherence to medication. N Engl J Med.

